# Supplementary material for: Are carbon emissions trading and green financial instruments synergistic? -Comprehensive quantitative research based on content analysis
Source: PLoS One. 2024 Mar 7;19(3):e0298601. doi: 10.1371/journal.pone.0298601 (PMC10919601; doi:10.1371/journal.pone.0298601)
Supplement: S1 Data — (ZIP) [file pone.0298601.s001.zip › Data/地级市Z_F工作报告.zip!/║╙▒▒/║Γ╦«╩╨/└┤╫╘╣1⁄2╓┌║┼í╛┬φ┐╦╩2╛▌═°í┐_2017.docx]

各位代表：

现在，我代表市人民政府，向大会报告政府工作，请予审议，并请市政协委员和列席会议的同志提出意见。

一、本届政府工作回顾二号首长

市五届人大一次会议以来的四年，是衡水发展史上极为重要、极不寻常的四年。在省委、省政府和市委的正确领导下，在市人大、市政协的监督支持下，在社会各界的通力协作下，我们紧紧围绕“强势开局、跨越赶超，全面建成小康社会”总要求，坚持稳中求进工作总基调，统筹做好稳增长、促改革、调结构、治污染、惠民生、防风险工作，攻坚克难、锐意进取，圆满完成各项目标任务，为如期全面建成小康社会奠定了坚实基础。

全力以赴上项目、稳增长，综合实力显著增强。全市经济发展持续保持“增速领先、总量进位”态势。四年来，地区生产总值由1011亿元增长到1413.4亿元，年均增长8.2%；全部财政收入由100.8亿元增长到173.2亿元，年均增长14.5%；一般公共预算收入由50.7亿元增长到95.7亿元，年均增长17.2%；累计完成固定资产投资4048.8亿元，年均增长19.2%。实施省市重点项目527个，完成投资1731.05亿元，新上亿元以上项目1237个，海伟石化新材料、阳煤乙二醇、正大现代农牧全产业链项目投资均超百亿元。地区生产总值、全部财政收入、一般公共预算收入、规模以上工业增加值、固定资产投资、社会消费品零售总额、进出口总值、出口总值、农村居民人均可支配收入9项指标摆脱了全省垫底位置，综合实力跃上新台阶，经济社会发展跨入新阶段。

千方百计转方式、调结构，经济发展提质增效。主动适应引领经济发展新常态，积极推进供给侧结构性改革，产业结构调整取得新进展，三次产业比重由2012年的18.7:51.7:29.6调整为2016年的13.0:46.9:40.1。现代农业强市建设步伐加快。粮食综合生产能力稳定在35亿公斤以上，畜牧、蔬菜、果品三大产业稳步提升，注册家庭农场6724家、农民合作社13812家，建成国家级现代农业示范区1家、省级现代农业园区12家，经营收入超亿元龙头企业24家，农业产业化经营率达到68.8%。工业转型升级跃上新台阶。大力改造提升食品医药、纺织家居等传统产业，积极培育新材料、高端装备制造等战略性新兴产业。工业技改投资逐年增加，企业管理水平不断提升，规模以上企业新增318家达到1196家，年纳税超千万元企业由44家增加到75家。现代服务业进一步壮大。旅游总收入突破百亿元，四年增加68.3亿元，年均增长24.9%。衡水湖景区、武强年画博物馆晋升4A级景区，衡水湖成功创建国家生态旅游示范区，周窝音乐小镇被评为中国乡村旅游创客示范基地。安平、武邑被列为国家级电子商务进农村综合示范县，饶阳被列为国家级一二三产业融合发展示范县。安平国际丝网物流聚集区、故城冀德物流园晋升省级物流产业聚集区。安平丝网、大营裘皮荣登2016年全国区域品牌价值百强榜。总投资40亿元的中铁物流园落户衡水，冀通快递物流园、饶阳新发地农副产品物流园、深州安华国际物流园等顺利推进。

多措并举抓统筹、搞建设，新型城镇化进程明显加快。以建设京津冀重要节点城市为引领，确立了近期“一湖三区”、远期“一湖六区”组团式发展思路和“四个一百”的发展目标。主城区同步推进滏南新区建设和河东旧城改造，滨湖新区有序推进新城建设，冀州顺利撤市设区，结束了一市一区的历史，市辖区面积扩大到1509平方公里，城市框架进一步拉大。全长120公里的城市高速外环、衡德高速故城支线、106国道衡水湖段改线等工程建成通车，石济客专即将通车，京衡高铁等工程加快推进，衡水区位交通优势进一步彰显。前进大街、榕花大街等全线贯通，启动了京衡大街改造提升和南外环贯通工程，改造提升135条小街巷，地下管网改造、地下垃圾池取缔、便道砖铺装、天然气路北环网、集中供热一次网互联等工程顺利推进，文化艺术中心、奥林匹克体育中心、市民中心等一批城市公共设施加速建设，城市功能日益完善。马拉松广场、衡百国际、怡然城、哈院综合楼、衡水学院新校区、衡水第一中学等项目投入运行，建成全国首批、全省首个“全光网城市”，主城区实现无线WIFI公共场所室外全覆盖，园博园、植物园、宝云文化公园等项目开工建设，城市品位不断提高。实施县城建设三年攻坚，坚持新区建设和老城改造两手抓，深入推进产城教融合发展，“多规合一”试点顺利推进，“六个一”工程取得新突破，县城承载力进一步提升，建成9个省级园林县城，枣强县荣获省十佳洁净城市。10个乡撤乡建镇，打造了一批旅游小镇、文化小镇、电商小镇。农村建设水平显著提高。完成938个美丽乡村重点村升级改造，打造了绳头、侯店、清辉头、端午、纪庄、白草洼等一批特色村。推进农村新型社区与产业园区、生态园区“三区同建”，启动农村新型社区61个，累计完成投资32亿元，枣强县八里庄新型社区列入省首批低碳社区试点。建国镇被农业部评为全国一村一品示范村镇。全市城镇化率由41.4%提高到48.9%。

攻坚克难推改革、促开放，发展活力不断增强。围绕国家中小城市综合改革试点和省级综合配套改革试验市，推出一批标志性、支柱性改革措施。农业农村改革走在全省前列。土地流转率、3000亩以上经营典型、注册家庭农场、农民合作社数量均居全省首位。试点推行工业用地供应先租赁后出让制度。桃城区“一提一补”节水机制在全国推广。金融生态进一步优化，交通银行、浦发银行、河北银行、邢台银行、广发证券等28家金融机构入驻我市。上市企业由5家发展到31家。建立市县两级投融资平台，全面推行债权银行合作公约制度。全面完成省定开发区改革试点任务。积极扩大对外开放。连续成功举办衡水湖国际商务洽谈会，北京蒙牛、泰国正大、日本积水等一批国内外500强企业落户衡水。衡水海关开关运行。故城、枣强、安平晋升国家外贸转型升级专业型示范基地。京津冀协同发展开局良好。明确了“一枢纽、四基地”功能定位，大力实施“三个一百”工程，“蓝火计划”衡水基地正式挂牌。工业新区被授予“中国新型工业化产业示范基地”，并被列入全省10个重点战略平台，滨湖新区被列入全省第一批承接非首都功能疏解“微中心”。全市与京津洽谈引进项目374项，总投资4138亿元，北京威克多制衣、上好佳天津大湖等一批企业整体搬迁我市。冀州游乐设备产业园、枣强高新技术产业园、武邑京津冀协同发展产业园、深州京津冀产业协作园、饶阳高新技术产业园、景县智能物流装备产业园、阜城食品产业园等加快建设。创新创业成效初显。中小企业创业辅导基地达到26家，市场主体由11万户增加到23.6万户。与中科院共建中科衡水科技成果转化中心，建设科技大市场，培育了原动力、衡智众创空间、蓝火众创空间等一批国家级、省级众创空间，院士工作站达到10家，省级工程技术研究中心达到16家。高新技术企业、科技小巨人企业、科技型中小企业分别达到124家、166家和3100家。

重拳铁腕治污染、保环境，生态优势持续放大。建成智慧环保管理平台，探索实行“双网格化”管理机制，制定出台排污证“一证式”管理办法，实行环保诚信黑名单制度，有效解决了“看不见、管不住”的问题。空气质量持续改善。紧紧锁定“煤、气、尘、车”四大主要污染源，先后开展了“工业企业污染排放专项整治”、“环保攻坚十大战役”、“净大气、抑扬尘，确保蓝天蓝”和大气污染防治行动计划等重大行动，去年我市空气质量达标天数130天，比2013年增加60天；PM2.5平均浓度87微克/立方米，较2013年下降28.7%。水环境持续好转。着力推进“一湖九河”重点流域污染整治、污水处理厂达标治理等工程，域内河流水质达到省生态补偿考核标准。2015年我市水污染防治工作被评为全省第一。抓好地下水超采综合治理试点，着力实施“节引蓄调管”五大工程，争取资金76.68亿元，形成6.21亿立方米压采能力，全市深层地下水平均埋深较试点前回升5.89米。不断深化拓展“一人一亩林”工程。全市造林面积达到480万亩，森林覆盖率达到30.1%，衡水湖森林公园、枣强欣苑、饶阳留楚等10个林业生态观光项目单体规模超万亩。

尽心竭力惠民生、为百姓，人民群众福祉持续增加。脱贫攻坚成效明显，59.1万贫困人口稳定脱贫，389个贫困村出列，枣强县摘帽。坚持每年办一批惠民利民的好事实事，民生支出由2012年的120.2亿元增加到244.3亿元。城镇累计新增就业17.1万人。城乡居民人均可支配收入分别达到23787元、10069元。城镇、农村低保标准比2012年分别提高53.1%、64.6%。南水北调中线配套工程完工，主城区居民喝上了长江水，323万农村人口解决了饮水安全问题。全面实行城乡免费义务教育，全市新改扩建中小学603所。衡水中学、衡水二中等普通高中教育水平不断提升，高等教育稳步发展。城乡居民基本医疗保险制度整合基本完成，扩大门诊救助病种范围，8所城市公立医院推行药品零差率销售，市四院、市中医院晋升三级医院。滏阳小学“童谣传唱社会主义核心价值观”活动得到刘云山等中央领导批示。法帖拓印技艺、硬木雕刻等列入国家、省非物质文化遗产保护名录。深化拓展“善行河北·厚德衡水”品牌，加快推进县城馆院和农村文化大院等公共文化设施建设，衡水中国书画博物馆入选中国十大民营博物馆。创作了《火种》《福星临门》等具有衡水印迹的文艺精品。连续五年成功举办衡水湖国际马拉松赛，连续四届获得金牌赛事称号，今年获得国际田联铜标赛事。成功举办首届花田音乐生活节。平安衡水建设扎实推进，食品药品安全监管力度不断加大，治安、消防和交管技术装备水平显著提升，安全生产形势总体平稳，社会大局和谐稳定。国防动员、双拥共建和优抚安置深入开展，军政军民团结局面进一步巩固。人口计生、民族宗教、新闻出版广电、外事侨务、涉台事务、人民防空、气象、防震减灾、地理信息、邮政通信、援疆、妇女儿童、老龄、残疾人、史志、档案等工作都取得新成绩。

坚定不移转职能、优服务，政府自身建设全面提升。深入推进“放管服”改革，“四个清单”制度全面建立，在全省率先实施“三证合一”，全部取消非行政许可审批事项，市本级行政审批事项取消下放691项，取消下放比例达到83.8%，市县实现行政审批局全覆盖，运行了几十年的近1500枚审批公章退出了历史舞台。以阜城县为试点，组建综合行政执法局，变“九龙治水”为“一支队伍管执法”。大力推进“三级平台”“两个代办”，在全市118个乡镇4038个村建立了便民服务站点。建立法律顾问制度，政府决策科学化、民主化水平不断提高。拓宽群众诉求渠道，开通961890群众服务热线，累计解决群众反映问题23万余件。全面落实省委、省政府和市委各项决策部署，自觉接受人大、政协监督，人大代表建议、政协提案办结率均达到100%。严格执行中央“八项规定”，认真落实党风廉政建设主体责任，巩固党的群众路线教育实践活动成果，扎实开展“三严三实”专题教育和“两学一做”学习教育活动。坚持依法行政，“六五”普法全面完成，政府法治意识和治理能力进一步增强。

各位代表！过去一年，面对宏观经济下行压力不断加大和环境约束日益趋紧的严峻形势，全市人民在省委、省政府和市委的正确领导下，凝心聚力，砥砺前行，保持了经济持续健康发展、民生不断改善、社会和谐稳定的良好势头，实现了“十三五”发展的良好开局。全市生产总值增长7.8%，规模以上工业增加值增长5.3%，固定资产投资增长13.4%，全部财政收入增长6.1%，一般公共预算收入增长8.1%，城镇居民人均可支配收入增长10%，农村居民人均可支配收入增长11.5%。11项指标增速超过全省平均水平，其中，地区生产总值、固定资产投资、社会消费品零售总额、城镇居民人均可支配收入、农村居民人均可支配收入等8项指标增速位居全省前三位。

各位代表！本届政府任期的四年，我们不甘落后、跨越赶超，是全市经济社会发展最好最快的时期，是城乡面貌变化最大的时期，是人民群众得到实惠最多、幸福感提升最快的时期，是我市地位形象、知名度、美誉度大幅提升的时期。我市经济社会发展取得的成就，是省委、省政府和市委正确领导的结果，是全市人民艰苦奋斗、锐意进取、共同努力的结果。在此，我代表市人民政府，向全市人民，向人大代表、政协委员，向各民主党派、工商联、无党派人士和人民团体，向驻衡部队、武警官兵和政法干警，向中直、省直驻衡各单位，向关心衡水发展的各界人士，致以崇高的敬意和衷心的感谢！

各位代表！回顾过去，成绩令人振奋，经验弥足珍贵。我们深切体会到，推进衡水经济社会又好又快发展，必须牢固树立“四个意识”，紧密团结在以习近平同志为核心的党中央周围，坚持以习近平总书记系列重要讲话精神引领航向，坚决有力地贯彻落实党中央国务院、省委省政府和市委各项决策部署；必须始终坚持以人民为中心的发展思想，统筹城乡发展，全力保障和改善民生，不断增加民生福祉；必须紧紧扭住发展这个第一要务，践行新理念，加快调结构转方式，提升发展质量效益；必须不断解放思想，向改革要动力、向创新要活力、向开放要空间，积极改善发展环境，凝聚绿色崛起、跨越赶超的强大力量；必须大力发扬“人一之我十之、人十之我百之”的作风和干劲，夙兴夜寐、激情工作，不断叫响“向落后说不、向倒第一说再见”，着力转作风、强素质、提效能，用实力、实绩、实效推进各项事业向前发展。

我们也清醒看到，我市经济社会发展中还存在不少困难和问题。经济总量小、发展水平低，传统产业发展面临瓶颈，新兴产业培育不足，科技创新能力不强，新旧动能转换不到位。城乡建设相对滞后，改善农村人居环境任务艰巨。生态环境建设形势严峻，大气污染治理措施需要进一步加强。全面深化改革氛围不够浓厚，发展环境仍需进一步优化，对外开放亟待加强。民生改善尚需付出更大努力。一些地方和部门懒政怠政、担当和服务意识不强的问题仍然存在。对此，我们一定高度重视，以民之所望为政之所向，积极面对，认真解决，决不辜负组织的重托，决不辜负全市人民的期望！

二、今后五年工作的总体要求和目标任务侯卫东官场笔记

当前，衡水改革发展已经站在新的历史起点上。今后五年，是我市推进供给侧结构性改革、实现新旧动能转换的关键时期，是推动发展路径转变、全面跨越提升的关键时期，是补齐发展短板、与全国全省同步全面建成小康社会的关键时期。衡水发展面临着难得的历史机遇，也存在着诸多挑战。世界经济增长低迷态势仍在延续，“逆全球化”思潮和贸易保护主义倾向抬头，国内经济下行压力依然较大。我们更要看到，我国发展仍处于大有可为的重要战略机遇期，经济发展新常态为我们“弯道取直”创造了新的空间；经过多年发展，全市经济体量不断扩大，综合竞争优势持续提升，跨越赶超的基础更加坚实；京津冀协同发展进入全面实施阶段，随着交通互联互通、产业协作发展、生态共建共享的深入，以及雄安新区的建立，必将极大提高衡水的创新能力和产业层次；综合配套改革向纵深推进，一系列重要规划和战略平台相继获批，一批高铁、高速、机场重大基础设施项目和百亿元、五十亿元、十亿元产业项目将相继建成投用，交通区位优势、县域经济优势、后发崛起优势进一步凸显，衡水正在进入由量的积累向质的跃升新阶段。市第四次党代会紧跟时代步伐，描绘了衡水未来五年发展的宏伟蓝图，激发了全市人民干事创业的昂扬斗志。只要我们保持战略定力、增强发展自信，乘势而上，拼搏进取，就一定能够掌握发展主动权，开辟发展新境界，谱写建设经济强市、美丽衡水的新篇章!

今后五年工作的指导思想是：全面落实党的十八大、十八届三中四中五中六中全会和省第九次党代会、省委九届二次全会及市第四次党代会精神，以邓小平理论、“三个代表”重要思想、科学发展观为指导，深入学习贯彻习近平总书记系列重要讲话精神，统筹推进“五位一体”总体布局，协调推进“四个全面”战略布局，认真贯彻创新、协调、绿色、开放、共享的发展理念，积极适应和引领经济发展新常态，大力推进供给侧结构性改革，按照赵克志书记对衡水提出的“三个努力”“四个突出抓好”要求，继续实施“项目立市、工业强市、三产兴市、农业升级、绿色崛起”发展战略，坚定走加快转型、绿色发展、跨越提升新路，为全面建成小康社会、加快建设经济强市美丽衡水而奋斗。

按照这一指导思想，今后五年我市经济社会发展的主要目标任务是：建设“一个高地”，实现“三个迈进”，打造“五个衡水”，实施“七大会战”，推动经济社会更高质量、更有效率、更加公平、更可持续的发展。建设“一个高地”，就是努力把衡水建设成为京津冀发展环境高地。实现“三个迈进”，就是迈进高铁时代、迈进智慧城市时代、迈进千亿产业时代。打造“五个衡水”，就是着力打造实力衡水、创新衡水、生态衡水、文化衡水、幸福衡水。实施“七大会战”，就是推进脱贫攻坚大会战、环境治理大会战、转型升级大会战、交通跨越大会战、园区建设大会战、城镇提升大会战、美丽乡村建设大会战。今后五年，要全面完成“十三五”规划的目标任务，主要经济指标增速高于全省平均水平，全市生产总值力争达到2000亿元，一般公共预算收入达到150亿元以上，进一步提升在全省发展大局中的地位和作用。未来的衡水，基础设施更加完善，产业结构更加合理，公共服务更加均等，各项保障更加健全，社会安定有序，人民安居乐业，我们将与全国全省一道，同步全面建成小康社会，实现第一个百年奋斗目标，开启第二个百年奋斗目标的新征程。

建成交通枢纽，再造区域发展新优势。石济客专、京衡高铁、石衡沧港城际建成通车，衡水实现京津石济一小时直达。石衡高速、衡港高速、任德高速衡水段建成通车，实现县域高速公路全覆盖，构建“一环、两纵、三横”高速公路网。改造提升国省干线和县乡村道路，完成市区公路大外环，谋划城市轻轨项目，形成市域内快速通勤圈，为城市组团发展奠定基础。加快推进衡水通用机场建设。基本形成以京衡、津衡、石衡、衡港、衡济五大运输通道为骨架，高速、高铁、航空为纽带，国省干道和县乡村公路为网络的现代化综合交通运输体系，实现与京津冀交通网全面对接，基本建成京津冀重要节点和冀中南综合物流枢纽城市。

建成千亿产业，壮大跨越赶超新动能。功能材料和食品医药成为超千亿元的支柱产业，工业新区主营业务收入超千亿元，努力创建国家级开发区。景州高新技术产业开发区、深州经济开发区、冀枣新材料基地、安平丝网工业园等主营业务收入超五百亿元。培育养元、老白干、正大、海伟、春风等5个超百亿元企业，支持泰华、宝力、中铁建、冀衡、瑞丰等一批竞争力强、成长性好、有社会责任感的民营骨干企业打造50亿元企业。文化旅游、大健康、现代物流、现代金融、电子商务、会议会展、楼宇经济、注册地经济等新产业、新业态蓬勃发展，力争衡水湖晋升5A级景区，衡水成为知名的文化旅游、运动休闲城市。服务业增加值占生产总值比重达46%以上，高新技术产业增加值占规模以上工业比重达22%以上，形成以现代农业为基础、先进制造业为主导、现代服务业为支撑、战略性新兴产业为引领的产业格局，为跨越赶超打造新引擎、注入新动能。

建成“四百”城市，构建城乡一体新格局。中心城市建成面积达到100平方公里、湖区水面100平方公里、环湖造林100平方公里、市区人口100万人以上，生态宜居滨湖园林城市初具规模。开展国家园林城市和省级文明城市、卫生城市、环境保护模范城市、食品安全城市“五城同创”，让城市功能更完善、环境更优美、品位更时尚、社会更文明。“一湖九河”大水系格局基本形成，建成园博园、植物园、奥林匹克体育中心等重点项目，加快河东旧城区、滏南新区、高铁商务区、宝云生态城、滨湖新城、冀州东部新区等片区建设，将枣强、武邑纳入同城化管理，稳步推进撤县设区，实现“一湖六区”协调发展。推进景县等撤县建市。加快县城建设，因地制宜打造一批各具特色、富有活力、宜居宜业的文化旅游、商贸物流、现代制造特色小镇，加快“三区同建”步伐，美丽乡村实现全覆盖，全市常住人口城镇化率达到60%以上，走出一条城乡统筹发展的新路子。

建成环境高地，激发改革开放新活力。全面完成综合配套改革各项任务，重点领域和关键环节取得一批创新性成果。优化政务、营商、人文、司法、舆论、数据、生态等环境，打造发展环境高地，促进各类市场主体公平竞争，提升经济开放度。着力推动协同创新，加大与京津高等院校、科研院所、科技企业对接合作，承接建设一批研究院、工作站和科技成果转化基地，吸引一批衡水学子回乡创新创业。坚持创新驱动战略，企业、产业、县域创新能力大幅提升，创新型企业和产品不断涌现，科技进步对经济增长的贡献率大幅提高。企业研发中心和研发投入强度高于全省平均水平，衡水进入创新型城市行列。

建成全面小康，谱写幸福和谐新篇章。脱贫攻坚任务圆满完成，现行标准下农村贫困人口实现脱贫，贫困村全部出列，贫困县全部摘帽。城乡居民收入增长高于经济增长。城乡环境大为改观，生态环境持续好转，绿色低碳循环发展模式初步建立，尽快退出全国空气重污染城市行列，天蓝、地绿、水秀的美丽衡水初步展现。惠及全民的公共文化服务体系基本形成，文化软实力和影响力持续提升。进一步做强高中教育品牌，打造职业教育高地，不断提升高等教育水平，吸引京津重点高校来我市联合办学。社会事业加快发展，社会保障实现全覆盖。法治建设进一步加强，社会治理水平明显提高，社会更加和谐稳定。社会主义核心价值观更加深入人心，全社会向上向善的氛围更加浓厚。社会矛盾化解成效显著，安全生产形势持续平稳，人民群众安全感和满意度不断提升。

实现上述目标，必须高举发展大旗，坚定不移用新发展理念指导实践、推动工作，坚持以供给侧结构性改革为主线，以改革统揽全局，以创新增强动力，走好“加快转型、绿色发展、跨越提升”发展新路。

（一）以创新引领发展，大力培育经济发展新动能。大力实施创新驱动战略，积极开展技术、管理、发展模式创新。用好京津创新资源，与京津共建科技园区、创新联盟等服务平台，着力打造北京科技成果转化中试基地。深入实施“中国制造2025”行动纲领和“互联网+”行动计划，以技术创新和管理创新改造提升传统产业。抓好科技型中小企业和高新技术企业发展，打造更多的小巨人企业和领军型企业。大力培育高端装备制造、电子信息（大智移云）、节能环保、大健康新医药等战略性新兴产业。深入推进大众创业、万众创新，发展众创、众包、众扶、众筹，催生新业态、新模式。依托衡水湖，加快发展金融、教育、运动休闲等现代服务业。以推动全域旅游和产业融合发展为目标，大力发展文化旅游业。以中铁物流园、冀通快递物流园等项目为抓手，打造全国商贸物流基地。到2021年，三次产业比重调整到10.5：43.5：46。

（二）扎实推进新型城镇化，积极构建城乡发展新格局。坚持城乡一体、全域衡水，完善各项规划，打造以主城区为核心的城镇空间布局。围绕“四个一百”目标，坚持组团式发展，做大做强中心城市，全面提升承载能力和辐射带动力，提高建设管理水平，使城市既有“面子”、更有“里子”。实施“小县大县城”战略，利用3-5年的时间使县城规模等级、容貌环境、承载聚集、居住条件全面改善。扎实推进小城镇建设。以环境美、产业美、精神美、生态美为要求，推进美丽乡村建设和“三区同建”。加快户籍制度配套改革，深入实施居住证制度，切实保障进城农民工权益。加快水电路气讯等基础设施向农村延伸，建立健全城乡统一的就业、教育、文化、医疗、社保服务体系，推进公共服务均等化。

（三）全面深化改革开放，不断增强内生发展动力。坚持服从服务大局、主动融入大局，在对接京津、服务京津中加快发展自己。积极对接“一带一路”战略，加快“走出去”步伐。抓好工业新区海关监管场所等申报建设，提升安平、故城、大营等国家外贸转型升级专业型示范基地，创新跨境电商等外贸方式，提高外贸规模和质量。积极争取将天津自贸区政策向我市扩展和复制。全面推进国家中小城市改革试点和省级综合配套改革试验市工作，积极争取国家和省改革试点，坚持每年推出一批“１+１”改革举措。全面深化农业农村改革、社会体制改革和生态文明体制改革。我们一定要让企业和群众更多感受到全面深化改革的成效，持续唱响走遍千山万水、投资就到衡水。

（四）加大环境治理力度，努力建设天蓝地绿水秀的美丽衡水。认真贯彻落实河北省大气污染综合治理大会精神，把生态环境建设作为战略性、全局性、根本性的重大问题，着力补齐生态环境短板，提高环境承载力。下大力抓好关于强力推进大气污染综合治理的意见和18个专项实施方案的落实，围绕“三下降、一增加”目标，科学治霾、协同治霾、铁腕治霾，把压煤减排、降尘增绿作为主攻方向，把调整结构、转型升级作为治本之策，把联防联控、执法监管作为有效途径，强化专项攻坚治理，动员全党全民全社会力量，下硬功夫、苦功夫、巧功夫，坚决打好蓝天保卫战。加强地下水超采综合治理，压采率达到85%以上，地下水基本实现采补平衡。www.libaiwu.com 政府工作报告。坚持绿化美化香化“三化”并举、乔灌花草整体推进，打响全域绿化攻坚战，大力提升绿化标准和质量，实现“一次植树、一次成林、一次成景”，全市森林覆盖率达到36%以上。加强衡水湖生态保护与修复，确保常年稳定在地表水三类水质标准，为子孙后代看护好衡水湖这片湿地。全面开展河渠整治，确保“一湖九河九渠”水质达到省考核要求。大力发展循环经济，打造近零碳排放示范区。逐步完善生态保护补偿机制和环境信息公开制度。强化环境执法监管，完善环保行政执法和刑事司法衔接机制。要把生态考量放在第一位，工业园区位置和工业企业选址，决不能摆在城市水源地附近和上风上水区域。桃城区、滨湖新区、冀州区坚决禁止新上化工项目，存量化工项目要向绿色化、精细化、零排放方向发展。

（五）坚持执政为民理念，致力满足群众生活新期待。深入实施精准扶贫精准脱贫，确保贫困县如期摘帽，贫困人口脱贫不返贫，确保脱贫成效经得起历史检验。多渠道增加就业岗位，建立覆盖城乡的就业创业服务体系。深化医疗卫生体制改革，让广大群众享受更方便、更快捷的医疗卫生服务。加强和创新社会治理，严格落实安全生产责任制，实施食品药品安全放心工程，确保社会和谐稳定。加快优质教育、文化、体育、养老等资源的优化布局和有效供给，形成覆盖城乡、群众共享的公共服务体系，不断提升人民的幸福感、获得感。

各位代表，目标任务已经明确，美好蓝图已经绘就。我相信，只要我们同全市人民一道戮力同心、砥砺奋进,就一定能够书写好“赶考”路上的衡水答卷！

三、2017年主要工作

今年是实施“十三五”规划的关键年，是全面落实省市党代会精神的第一年，也是新一届政府的开局之年，做好今年工作意义重大。综合考虑各方面因素，今年全市经济社会发展的主要预期目标是：地区生产总值增长8%以上，规模以上工业增加值增长7%左右，全部财政收入增长10%左右，一般公共预算收入增长10%左右，固定资产投资增长13%左右，社会消费品零售总额增长10%左右，出口总值增长3%以上。PM2.5平均浓度、单位生产总值能耗、化学需氧量、二氧化硫、氨氮和氮氧化物减排等指标完成省下达的任务。城镇、农村居民人均可支配收入分别增长10%和11%左右。居民消费价格指数涨幅控制在3%左右。城镇登记失业率控制在4.5%以内。具体要紧紧围绕市委确定的“七个突破年”，做好以下九项工作：

（一）全力推进项目建设。始终把项目建设作为经济社会发展的总抓手，着力提高项目谋划、争取和实施能力，形成大上项目、上大项目、上好项目的浓厚氛围。

狠抓一批重大项目。以“323”工程为抓手，完善“三个二”项目推进机制，抓好省市重点项目300项，新开工亿元以上项目200项，推进市政重点工程30项，力争中广核生物天然气、舜鑫现代智能物流装备、烨和祥生物玉米纤维、中仪联众高铁智能轨道系统等55个续建项目建成投产，力促万德康自动回缩式注射器、中京轨道交通智能化电气装备等85个项目尽快开工，争取中铁物流园、德隆国家装配化产业示范区、冀德现代综合物流等前期项目提前启动。抢抓国家政策机遇，围绕补短板、调结构、促创新、惠民生，精心谋划未来五年的战略支撑项目，力争每年新谋划10亿元以上项目100项以上。

精准开展招商引资。提高招商层次。强化与国家部委、京津相关部门及区县的高层精准对接，实施“百家央企名企进衡水”活动，围绕特色产业链条延伸、科技成果转化合作、新型消费热点培育等领域，以最大的决心和诚意引进一批大项目好项目。改进招商方式。提升专业化招商水平，整合共享招商引资信息，制定更有竞争力、更有针对性的招商政策，做好小团组招商、以商招商、驻点招商、网络招商、代理招商、亲情招商、产业链招商。坚持招商与招才并举、引资与引智并重，力争引进一个项目、带来一批人才、打造一个团队、搞活一个产业。注重招商实效。提高签约项目的履约率、资金到位率和开工率。各县市区主要领导要亲力亲为，每县都要新谋划引进一个超百亿元项目。在组织好“5·18”廊洽会、衡水湖国际商务洽谈会的同时，今年重点抓好与北京顺义区承接产业转移对接活动、与京津等高校（科研院所）合作对接恳谈活动。全面落实与河北工业大学的战略合作协议。

强力实施园区攻坚。编制衡水市开发区总体发展规划，进一步明确全市各类开发区发展方向，促进形成布局合理、主业突出、特色鲜明、错位发展、协作配套的发展格局。以做优生态、完善基础设施和配套服务功能为重点，提高承载能力。年内，所有省级园区建成区实现“九通一平”和WIFI全覆盖，起步区实现高水平的“新九通一平”。全面深化开发区体制机制改革，年内省级开发区全部建立市场化运作的专业招商公司，推动产业集聚和布局优化，打造项目建设的主战场、创新创业的主平台。年内，全市省级开发区、高新区主营业务收入达到3000亿元以上，固定资产投资、工业增加值、税收增长15%以上。

（二）促进产业转型升级。以推进供给侧结构性改革为主线，深入实施创新驱动发展战略，不断提高实体经济的质量、效益和竞争力。

推动传统产业提档升级。实施增强制造业核心竞争力三年行动计划，推动两化深度融合，加快新一轮技术改造，推进智能工厂、数字化车间建设。严格执行环保、能耗、质量、安全等法律法规和标准，更多运用市场化法治化手段，有效处置“僵尸企业”，坚决打破拖累衡水发展的坛坛罐罐。扶优做强骨干企业，带动特色产业转型升级。食品医药产业，重点向方便休闲食品、绿色有机食品、生物工程食品和高端生物制品方向发展。支持养元公司完善产品链、品牌链，进一步扩大市场占有率，再造新品牌。全面启动老白干酒业改制，搞好顶层设计，加快并购重组，彻底放活机制，力争2-3年内做成百亿元企业。功能材料产业，重点向先进高分子材料、高性能复合材料及制品方向升级。依托泰纳、海伟等企业，打造国家级功能材料产业基地。装备制造产业，加快由零配件、器材、功能部件向整体装备方向迈进。支持泰华加速研发新技术、新产品，向水下推进器、无人机飞控系统、3D打印等领域发展。依托卡斯卡特、博索尼等企业，引进叉车整车生产，提升装备制造水平。依托鹤煌、金普信等企业，提高加工精度和深度，推动丝网产业向中高端迈进。依托宝力、中铁建等企业，拓展产品领域，提升制造水平，壮大工程橡胶等交通装备产业集群。纺织家居产业，重点向高端化、品牌化、特色化、绿色化方向发展。依托威克多、华兴、顾家、安德森等企业，推行个性化定制、服务型制造等新模式新业态，增加高品质、功能性、智能化产品比重。深入开展帮扶企业活动，进一步清费减负，积极帮助企业去杠杆、降成本，推动“个转企、小升规、规改股、股上市”，年内新增规模以上工业企业100家以上。

积极培育战略性新兴产业。加快发展高端装备制造、新材料、新能源、电子信息、节能环保、文化旅游、大健康新医药等产业，打造战略性新兴产业高地和高新技术产业集群。引导人才、技术、资金、土地等要素向战略性新兴产业聚集，每个产业都要重点引进培育2-3家领军企业，打造具有衡水特色的新兴产业发展格局。

不断拓展现代服务业。推进中铁物流园、冀通快递物流园、肖张商贸物流园等一批物流园区建设。推动全域旅游和产业融合发展，围绕建设一批旅游新业态项目，打造一批重点旅游片区。冀州区、武强县要全力创建省级全域旅游示范区。依托自然景观、历史古迹和文化资源等，打造衡水湖汉民俗文化小镇、周窝音乐小镇、广川董子文化小镇等一批特色旅游小镇。大力发展健康养生、运动休闲、特色教育、社区服务等生活性服务业，积极培育信息服务、工业设计等新业态新模式，打造“智慧衡水”。

全面提升质量品牌。坚持以质取胜、品牌兴市，全面开展“质量提升年”活动，深化标准、质量、品牌一体化建设，逐行业开展质量提升行动，主动参与行业标准制定，谋划建设一批国家级质量检验检测中心，抢占行业制高点，用质量优势对冲成本上升，以质量先行赢得发展主动。速度终有上限，质量永无止境。我们要牢固树立质量第一的强烈意识，始终保持追求卓越的质量自觉，恪尽职业操守，崇尚精益求精，培育更多“衡水工匠”，打造更多“衡水品牌”，推动衡水经济发展进入质量时代。

（三）加快建设现代农业强市。从农业供给侧结构性改革入手，培育农业农村发展新动能，提高农业综合效益和竞争力，着力增加农民收入和有效供给，努力让农业强起来、农民富起来、农村美起来。

提升农产品供给质量。紧紧围绕市场需求发展生产，调优种养结构，调强加工能力，调大经营规模，调长产业链条，加快形成结构更加合理、保障更加有力的农产品供给体系。按照“稳粮、强奶、扩畜、培特”的思路，加快构建粮经饲协调发展的三元结构。全市粮食综合生产能力稳定在35亿公斤以上，适度调减籽粒玉米种植规模。实施奶业振兴示范市建设行动计划，年内75%的奶牛养殖场（区）完成标准化改造。按照“一县一业一品一特色”的思路，加快建设京津绿色农产品供应基地。全面推进农产品质量安全示范县创建，年内农业标准化生产覆盖率达到50%以上。积极推进农村一二三产融合发展，做大做强现代农业园区。年内争创省级现代农业园区6个，力争把京安集团建成国家农业可持续发展试验示范区，培育2－4个在全省领先、全国挂号的现代农业园区。推广正大集团种养加一体化全产业链模式，谋划实施投资千万元以上农业产业化项目100个以上。实施品牌农产品推介行动计划，打造一批在全省、全国乃至国际上有影响力的行业企业品牌、区域公用品牌和旅游农产品品牌，把衡水打造成农产品品牌的聚集区、优势区。

强化科技金融支撑。稳定基层农技推广队伍，全面实施农业科技入户、入场、入园区工程，全方位开展农业科技服务。注重农耕文明的传承，多层次、多渠道开展农业科技培训，培育现代职业农民。全面推进基层党组织与基层农村信用社“双基”共建农村信用工程。推广饶阳“政银企户保”经验，通过政府搭台增信、银行降槛降息、企业农户承贷、保险兜底保证，让金融机构风险最小化、农民利益最大化，加快农民致富和农村中小企业发展。

着力深化农村改革。积极稳妥推进“三块地”改革，完善农村土地“三权分置”办法，推进农村集体产权制度改革，发展农村股份合作制经济组织，年内每个村都要建一个股份制经济实体。积极培育农业新型经营主体，新增工商注册家庭农场500家，规范化农民合作社达到1300家，农民合作社联合社达到70家。

（四）扎实推进新型城镇化。着眼城乡一体化发展，实施城镇提升大会战，把城乡面貌提高到一个新水平。

加强城乡规划设计。完善规委会决策机制，增强城乡规划的科学性和权威性。因应行政区划调整，面向全球聘请知名设计团队，启动城市总体规划修编，统筹生产、生态、生活空间布局，协调推进“多规合一”，实现全市“一张图”管控。编制全市生态环保规划，划定城市开发边界和生态红线，最大限度留出生态空间。启动城市水系循环规划，本着“先循环、后行船”的要求，让城区水系“活”起来。注重城市设计和单体建筑设计，落实城市建筑适用、经济、绿色、美观的方针，提升城市风貌特色。保护和修缮有历史价值的建筑群落，传承历史记忆。加强城乡规划衔接和管控，县市区实施重大工程、重大项目必须严格规划审批。冀州全区上下要增强融入主城区意识，市直相关部门年内要出台同城管理办法。要把去库存与促进人口城镇化、棚户区改造和保障性住房建设结合起来，加快建立和完善促进房地产市场平稳健康发展的长效机制。

做大做强中心城市。以“两城两会一赛事”为引领，加快补齐短板、完善功能，完成高铁广场建设、文化艺术中心、火车站广场改造、京衡大街提升等工程，着力推进奥林匹克体育中心、南外环贯通、大庆路和人民路东延等重点工程，谋划实施动物园、儿童乐园、科技馆等公共服务设施项目，决不能让我们的孩子输在起跑线上。成立专班加快推进哈院新院区建设，争取今明两年完成主体建设，2019年投入使用。抓好市中医院新院区建设。加快推进河东老城区改造和冀州东部新区建设，打造高铁商务区和宝云生态城，加快谋划中央商务区建设，依托衡水中学等基础教育品牌，高标准规划建设教育园区。加大棚户区改造、地下管廊、停车场、停车楼等建设力度，谋划雨污分流改造，推动海绵城市、智慧城市建设取得突破性进展，提升中心城市承载能力。开展“五城同创”，以石济客专通车和省第二届园博会举办为契机，实施城市管理整治年，提升城市网格化、精细化管理水平，推动城市容貌和营商环境实现新跨越。重点打好违章建筑拆除、车辆乱停乱放整治、广告牌匾和主街道立面规范、城市绿化美化香化提升“五大战役”。年内，完成国家园林城市验收。

加强县城和小城镇建设。开展县城建设和环境综合整治攻坚行动，以城市修补、生态修复为导向，完善公共设施，补齐功能短板，推动产城教融合化、环境容貌整洁化、建设改造精品化，继续抓好“六个一”工程和9类公共服务设施规划建设。6月底，市县要全部完成城镇集中供暖专项规划编制工作，年内集中供暖和清洁能源供暖率达到75%以上。今年各县市全部创建成为省级园林县城。完善小城镇镇区道路、给排水、垃圾处理等公共基础设施和文化教育、卫生体育等公共服务设施。每个县市区重点抓好1-2个中心集镇，全市倾力打造9个省级重点特色小城镇。

开展美丽乡村建设大会战。按照新型城镇化与城乡统筹发展的理念，扎实推进美丽乡村建设。修订完善美丽乡村建设规划、全市村镇体系规划，与“四化同步”相结合，走“三区同建”的路子，加大村庄整合力度，打造一批宜居宜业宜游的美丽乡村综合体。各县市区每年至少培育3-5个“三区同建”示范点。以改厕改厨、能源替代为突破口，深入推进农村人居环境整治，建设既有现代文明、又具田园风光的美丽乡村。今年突出抓好1个省级重点片区、2个市级重点片区、500个省级重点村。

（五）深入推进改革开放。深化重要领域和关键环节改革，积极主动扩大对外开放，进一步激发发展活力。

继续深化改革。围绕使市场在资源配置中起决定性作用和更好发挥政府作用，持续推进政府职能转变，深化“放管服”全链条改革，着力加强“两局三中心”规范化、标准化建设，推行市县行政审批局同权划转、同步下放、同标运行。大力推进综合执法改革和城市执法管理体制改革，全面启动行政执法“三项制度”改革试点工作。推行“互联网+政务服务”，实行网上办事、网上服务、网上监管，形成线上线下相结合的政务服务新模式，加快建立全市统一的政务平台、支付平台、信息交换共享平台，让数据多跑路、群众少跑腿。深化财税体制改革，营造简洁透明、更加公平的税负环境。深入推进政府预决算公开，倒逼沉淀资金盘活，提高资金使用效率。深化混合所有制改革和供销合作社改革。支持各类企业发展直接融资，力争瑞丰、中科能源年内在港交所上市，推动养元、宝力尽快在国内主板上市。4个国家级贫困县要用足用好上市绿色通道政策，积极发展注册地经济。加快衡水银行、农村商业银行改革步伐，积极跑办引进外地金融机构。积极对接国开行、农发行、亚投行，用足用好政策性开发性贷款。毫不动摇鼓励支持民营经济发展，进一步拓宽市场准入，消除隐性壁垒，支持企业家专心创新创业。今年市本级和各县市区都要尽可能多地设立产业引导基金，用于支持技术改造、产业培育、品牌建设，促进科技成果转化。

积极扩大外贸出口。加大外贸支持政策落实力度，加强皮毛、纺织服装、丝网、机电产品等出口基地建设，帮助企业拓展市场。发展跨境电子商务，加大对外贸综合服务企业的支持力度，培育外贸发展新动能。

（六）积极推动京津冀协同发展。在对接京津、服务京津中加快补齐短板，培育发展新优势。

抓好政策精准衔接。加强与国家、省规划和一系列专项规划精准衔接，推进京津冀协同发展规划在我市落到实处。特别是抢抓国家设立雄安新区的重大机遇，超前谋划，深度对接，争取最大限度分享红利、承接辐射。抓紧研究天津自贸区政策在我市复制的路径办法。抓好重大承接平台建设。充分发挥工业新区和滨湖新区两大平台的引领作用，吸引京津和国内外创新资源，加速要素聚集和产业聚集。加快推进京衡客专、石衡高速、石衡沧港城际、衡港高速等重大基础设施建设。

抓好协同创新共同体建设。依托衡水中学等基础教育品牌优势，形成长效机制，开展与京津等著名高校、科研院所以及科技机构的深入对接合作，巩固“蓝火计划”成果，推动知名高校、创新团队、国家实验室、重点科研项目、军民融合项目等更多落户衡水，积极争取更多设立院士工作站、中试基地等机构。依托工业新区，全力建设衡水科技谷。加快推进桃城区航空飞行营地、中关村e谷衡水创业小镇、京津冀数字货币产业园的前期工作。依托滨湖新区，着力建设京津冀医疗卫生协同发展能力提升区和滨湖健康休闲养生产业园。

（七）持续抓好生态环境建设。牢固树立“绿水青山就是金山银山”的强烈意识，以建设京津冀生态环境支撑区为方向，大力度抓好环境保护，厚植生态优势。

坚决打好蓝天保卫战。坚持标本兼治、综合施策、重在治本，围绕尽快退出全国重污染城市行列目标，抓紧制定“1+27”一揽子意见措施，着力打好压煤、优企、控车、抑尘、增绿五大攻坚战，全面提升重污染天气应对能力和科技支撑能力，推动环境空气质量加快改善。下大力降低煤炭消费。加快推动“无煤区”建设，主城四区建成“无煤区”，其他县市建成区建成“高污染燃料禁燃区”。抓好农村散煤治理，坚持以气为主、气电并行，宜气则气、宜电则电、宜水则水，年内完成11.2万户煤改气工程，6月底前实现衡水全域禁售劣质散煤，存量劣质散煤统一回收、等量置换。在故城、深州、饶阳、安平、武强等有条件的县市大力开发地热能集中供热。工业新区加快推进“西汽东输”供热管网项目建设，替代关停企业锅炉。10月底前，市区建成区淘汰35蒸吨及以下燃煤锅炉，城乡接合部及县城淘汰10蒸吨及以下燃煤锅炉、茶炉大灶、经营性小煤炉。下大力整治工业污染。实施工业污染源全面达标排放行动计划，重点污染源实现稳定达标排放。今年净削减煤炭消费3万吨，完成5家工业企业搬迁改造。10月底前，全部完成“散乱污”企业整治任务。下大力控制机动车污染。全面强化货运车辆管控，实行入境货车远端绕行，完善公共交通服务体系，加快油品质量升级，加大新能源机动车推广使用力度。下大力整治扬尘污染。以道路扬尘、建筑施工、工业料场、秸秆垃圾禁烧等为重点，提升精细化、系统化、常态化治理水平。以改革创新的办法破解瓶颈难题，完善联防联控、应急响应、资金筹措等工作机制。引导企业主动履行社会责任，发动群众积极广泛参与，践行绿色低碳生活方式，少开一天车、少用一度电、多植一棵树、不用劣质煤、不乱烧垃圾秸秆等，形成人人有责、人人作贡献的浓厚氛围。严格执法检查和督导考核，推动大气污染防治尽快步入规范化、法治化、常态化轨道。积极应对重污染天气，提高应急响应的精准性和有效性，最大限度“削峰降频”。2017年全市PM2.5浓度力争比2016年下降14%左右，其中冬季取暖期比2016年同期下降18%以上。

实施碧水行动。在全市开展水污染防治百日攻坚战。全面推行市县乡村四级河长制，强化防汛抗旱责任制，构建责任明确、分级管理、监管严格、保护有力的管理保护机制。继续抓好“一湖九河九渠”综合整治，推进衡水湖西湖湿地恢复和北湖退田还湖、滏阳河生态修复、胡堂排干、班曹店排干等生态修复工程。加强地下水超采综合治理，压采率达到65%以上，城市地下水基本实现采补平衡，基本建立地下水取水计量、水位监测系统。大力实施空地一体化人工影响天气全覆盖工程。加快南水北调配套工程建设，加大江水切换力度，确保4月底江水用量达到切换前的供水量，年内达到规划分水量的30%以上，积极开展枣强、景县城乡供水一体化试点工作。

实施净土行动。开展土壤污染状况详查、土壤质量评估和类别划分工作，抓好畜禽养殖废弃物处理和资源化利用、危险废物治理、涉重金属企业清理整顿、关停搬迁企业污染场地治理修复等专项整治。在城乡大力推行垃圾分类。围绕林业园区、绿美廊道、环湖环城绿化、农村生态建设四大重点，用好融资平台，完善市场化造林新机制，打好增绿攻坚战，今年全市完成造林15万亩以上。

（八）统筹推进各项社会事业。我们将继续集中财力办好20件民生实事，努力在改善民生上见到更大成效。

打好脱贫攻坚大会战。扎扎实实抓好产业扶贫、就业创业帮扶、健康扶贫等工作，确保今年4万贫困人口脱贫、347个村出列，力争两个贫困县摘帽。完善财政扶贫资金增长机制，加大涉农资金整合力度，进一步落实贫困人口医疗保障救助政策，提高贫困人口医疗保障救助水平。

大力促进就业创业。实施高校毕业生就业创业促进计划，搞好农民工职业技能培训，支持农民工返乡创业，做好军队转业干部和退役士兵的接收安置工作，确保2017年城镇新增就业3.85万人，零就业家庭动态为零。

完善社会保障体系。继续完善“五险合一”征管机制，抓好企业养老保险和工伤保险的扩面征缴工作。依法将农民工纳入城镇职工基本养老和医疗保险，提高农业转移人口参加城镇社会保障比例。把进城稳定就业的农民工纳入城镇住房保障体系。

办好优质公平教育。推进新一期学前教育三年行动计划，抓好主城区中小学规划建设。大力发展现代职业教育，进一步深化产教融合，拓展职教园区功能，提升发展水平，培养社会急需的技能型人才。

发展文化事业和文化产业。认真践行社会主义核心价值观，深化拓展“善行河北·厚德衡水”主题道德实践活动。以创建省级文明城市为龙头，深入开展群众性精神文明创建活动，推进移风易俗，树立文明新风。推进现代公共文化服务体系建设，深入开展全民阅读，加强科学普及。培育壮大文化创意、节庆会展、演艺娱乐、出版发行等文化产业集群。加强文物和非物质文化遗产保护利用，推动中华传统优秀文化传承发展。高标准办好河北省第二届园博会暨全市首届旅发大会。广泛开展全民健身，按国际田联铜标赛事标准办好2017衡水湖国际马拉松赛暨全国马拉松锦标赛。

推进健康衡水建设。深入开展冀州、阜城县级公立医院综合改革示范县创建活动，探索建立县域内县乡一体化医共体，推行分级诊疗制度。支持中医药事业发展。建设市县两级人口健康信息平台，发放、应用“居民健康卡”，实现居民健康全生命周期管理。

（九）着力维护社会和谐稳定。强化源头治理、专项治理、系统治理、综合治理、依法治理，有效防控各类风险，提高社会治理水平。坚持以群众工作为统揽，开通网上信访平台，完善机制，主动作为，用心用情用力化解矛盾，依法及时就近解决群众合理诉求。健全立体化信息化社会治安防控体系，做好重大活动安保工作，严厉打击黑恶势力、电信网络诈骗等各类违法犯罪行为，严格规范文明执法，全方位提高人民群众安全感。加强金融监管，建立健全政府性债务风险应急处置机制，防范化解企业资金链、担保链风险，严厉打击非法集资活动，加强信用环境建设，确保不发生系统性、区域性金融风险。加快推进公共法律服务体系城乡一体化建设，促进公共法律服务资源向农村和基层延伸。扎实开展企业安全生产主体责任落实攻坚年和县域安全监管规范化建设活动，强力推进安全生产大检查和“打非治违”专项行动。开展对各县市区政府食品药品安全考核评价，建立覆盖农产品种植、养殖、屠宰、食品生产经营全链条的食品安全追溯平台。

加强国防教育和国防后备力量建设，促进军民融合深度发展，巩固军政军民团结。充分发挥工会、共青团、妇联等人民团体桥梁纽带作用。继续做好新闻出版广电、民族宗教、人口计生、外事侨务、涉台事务、人民防空、气象、防震减灾、地理信息、邮政通信、援疆、妇女儿童、老龄、残疾人、史志、档案等工作。

四、加强政府自身建设

各位代表，新形势新任务对政府工作提出了新要求，我们必须以新理念引领政府工作创新突破，努力建设法治政府、效能政府、服务政府、廉洁政府。

我们一定扛起责任，勇于担当。树立功成不必在我的理念，坚持一张蓝图绘到底，决不急功近利搞“形象工程”和“政绩工程”。增强克难攻坚的胆识，集中力量抓大事、破难题、啃硬骨头，看准的事情坚定干、抓到底，决不对分内的职责推诿躲闪。把政治责任转化为推动发展、改善民生的履职动力，做到日常工作能尽责，难题面前敢负责，出现过失勇担责，真正把使命和责任扛在肩头。

我们一定践行宗旨，俯首为民。坚持虚心向群众问计求教，广泛听取和及时回应群众的呼声诉求。坚持以增进百姓福祉为指南，在公共资源配置、市政工程安排、财政资金使用上优先向民生倾斜，满腔热情为群众办实事。自觉接受市人大及其常委会的法律监督、工作监督和市政协的民主监督及社会舆论监督，使政府工作更接地气、得民心，充分体现人民的意愿。

我们一定尊崇法治，依法行政。严格遵守宪法和法律，坚持运用法治思维、法治方式开展工作，坚决克服习惯性，减少随意性，杜绝盲目性。坚持政府及工作人员学法制度，推动法律顾问制度全覆盖。加强行政复议和行政应诉工作，依法行政，依法履职，依法决策，依法办事，真正做到尊法学法守法用法，努力建设法治政府。

我们一定实干实政，从严治政。认真落实全面从严治党要求，严格落实中央“八项规定”精神，坚决防止“四风”反弹回潮。深化机关作风整顿，各项工作都要高标准、严要求，大兴马上就办、办就办好的作风，夙兴夜寐、激情工作，做到“精致、细致、极致”。严格执行工作责任制，深入一线调查研究，察民情、接地气、出实招，以“钉钉子”的精神狠抓落实，撸起袖子加油干，确保圆满完成各项目标任务。强化督查问责，严厉整肃为官不为、懒政怠政行为。加强行政监察和审计监督。保持惩治腐败高压态势，严肃查处侵害群众利益的不正之风，严防“微腐败”成为“大祸害”，切实做到干部清正、政府清廉。

各位代表，实干托起梦想，奋斗铸就辉煌。全面建成小康社会，是新一届政府的光荣使命。让我们更加紧密地团结在以习近平同志为核心的党中央周围，在省委、省政府和市委的正确领导下，坚定信心、主动作为，不忘初心、继续前进，为全面建成小康社会，实现“经济强市、美丽衡水”的宏伟蓝图而努力奋斗！
